# Structured Polymers Enable the Sustained Delivery of Glucocorticoids within the Intra‐Articular Space

**DOI:** 10.1002/adhm.202403000

**Published:** 2024-12-23

**Authors:** Ana Crastin, Claire S. Martin, Sai Suresh, Scott P. Davies, Daniel Kearns, Ahsen Parlak, Holly Adcock, Andrew Filer, Simon W. Jones, Karim Raza, Richard JA Moakes, Liam M. Grover, Rowan S. Hardy

**Affiliations:** ^1^ Dept of Biomedical Sciences. Institute of Clinical Sciences University of Birmingham Birmingham UK; ^2^ Dept of Metabolism and Systems Science University of Birmingham Birmingham UK; ^3^ Centre for Liver and Gastrointestinal Research Institute of Immunology and Immunotherapy University of Birmingham Birmingham UK; ^4^ Liver Services Unit Queen Elizabeth Hospital Birmingham University Hospitals Birmingham NHS Foundation Trust Birmingham UK; ^5^ School of Chemistry University of Birmingham Birmingham UK; ^6^ Dept of Inflammation and Aging University of Birmingham Birmingham UK; ^7^ MRC Arthritis Research UK Centre for Musculoskeletal Ageing Research University of Birmingham Birmingham UK; ^8^ Healthcare Technologies Institute School of Chemical Engineering University of Birmingham Birmingham UK

**Keywords:** arthritis, gellan, glucocorticoid, intra‐articular, sheared hydrogel

## Abstract

Intra‐articular glucocorticoid injections are effective in controlling inflammation and pain in arthritides but restricted by short duration of action and risk of joint degeneration. Controlled drug release using biocompatible hydrogels offers a unique solution, but limitations of in situ gelation restrict their application. Gellan sheared hydrogels (GSHs) retain the advantages of hydrogels, however their unique microstructures lend themselves to intra‐articular application – capable of shear thinning under force but restructuring at rest to enhance residence. This study examined GSHs for extended intra‐articular glucocorticoid delivery of prednisolone (10 mg mL^−1^); demonstrating links between material mechanics, steroid release, and preclinical assessment of efficacy in synoviocyte culture and transgenic(TNF)197Gkl (TNFtg) murine model of arthritis. GSHs demonstrated sustained release, with typical Fickian profiles over 18 days. Moreover, systems showed good stability under extended culture, with inherent cell‐compatibility and suppression of inflammatory synoviocyte activation. In TNFtg animals, GSHs suppressed synovitis (70.08%, *p* < 0.05), pannus formation (45.01%, *p* < 0.05), and increased articular cartilage (82.23%, *p* < 0.05) relative to vehicle controls. The extended profile of steroid release from injectable GSH formulations holds promise in the treatment and management of inflammatory arthritides such as rheumatoid and osteoarthritis, representing a step‐change in intra‐articular drug delivery to suppress long‐term joint inflammation.

## Graphical abstract

Steroid injections are effective in diseases such as rheumatoid arthritis but limited by short duration of action and risks of joint degeneration with repeat application. It is demonstrated that steroids delivered in injectable non‐Newtonian Gellan sheared hydrogels are highly suited to this application, being cell‐compatible and possessing improved drug release profiles and anti‐inflammatory efficacy in a murine model of polyarthritis.

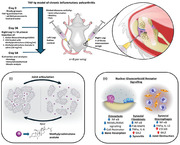

## 1 Introduction

Joint diseases such as rheumatoid (RA) and osteoarthritis (OA) result in progressive joint destruction characterized by chronic pain and disability.^[^ ^1^, ^2^ ^]^ While their underlying pathophysiology vary, joint inflammation and synovitis, with localized loss of cartilage and bone resulting in deformity are common features.^[^ ^2^ ^]^ While disease modifying interventions show efficacy in RA, management of OA frequently rely upon physiotherapy, pain management with non‐steroidal anti‐inflammatory drugs and ultimately, joint replacement. Both diseases are also widely managed with intra‐articular glucocorticoid injections to control pain, local swelling, and inflammation, given at intervals of at least three months.^[^ ^3^ ^]^ rapid elimination kinetics and necessitate multiple injections over the course of a year.^[^ ^4^ ^]^ These readily soluble solutions are rapidly lost from the joint into the wider circulation, and so to overcome this, formulations are administered at high doses, resulting in a potent but short acting therapeutic window that With high absorption, distribution, metabolism, and excretion (ADME), These formulations are rapid acting and effective in controlling pain but show limited efficacy in slowing disease progression and joint destruction.^[^ ^3^, ^5^ ^]^ Furthermore, the high doses required to overcome clearance within the joint are associated with off target side effects including increased risk of localized bone and articular cartilage loss, joint space narrowing, local muscle wasting, and an increased risk of infection that can contribute to joint degeneration and subchondral insufficiency fractures.^[^ ^6^, ^7^, ^8^, ^9^ ^]^ Despite these limitations, the efficacy of intra‐articular joint injections is such that they have received the highest level of endorsement in clinical practice guidelines, with a predicted global market value of $5.7 billion by 2026^[^ ^10^ ^]^ Therefore, there remains an unmet need to develop better tolerated, longer lasting injectable glucocorticoids that possess a reduced profile of off target side effects in cartilage muscle and bone within the joint.

Hydrogels, a swollen network of polymers capable of structuring vast volumes of solvent, hold much promise within controlled delivery owing to their highly tunable properties; for example, hydrophilicity, swellability, porosity, degradability *etc*.^[^ ^11^, ^12^ ^]^ Moreover, careful control over the polymer chemistry facilitates engineering of extended drug release profiles to maximize the therapeutic window, reduce the need for repeat injections, and eliminate high dose formulations that are associated with off target side effects. However, their application within the intra‐articular space is currently constrained by the route of administration; either requiring exogenous gelation and the implantation of pre‐formed gels through invasive surgical procedures, or in situ gelation.^[^ ^13^, ^14^ ^]^ As a non‐invasive approach, the latter offers a much more palatable option for medical treatment, applied in a low viscosity format through a needle and gelled in place (crosslinking stimulated using thermo‐/light‐sensitivity, pH, or ionic environment).^[^ ^13^, ^14^, ^15^ ^]^ Unfortunately, to date in situ approaches have been met with limited success: notably complications relating to therapeutic handling and delivery of complex polymer precursor reagents in a clinical setting, and inconsistency in uniform exposure to the gelling stimulus (pH, ionic composition, temperature) within the joint resulting in heterogenous gelation that effect drug release. These issues are compounded within the intra‐articular compartment, where formulations are subsequently exposed to continuous mechanical loading, stresses, and strains, causing fragmentation, debris (that can exacerbate OA), and resulting in uncontrolled drug release.^[^ ^16^ ^]^

Recently, fluid gels (also known as sheared hydrogels) prompted a new approach to minimally invasive technologies. These micro‐particulate suspensions, formed under shear during the sol‐gel transition, provide a unique microstructure that readily traverse solid‐liquid‐solid transitions at low forces without the need for chemical or thermal stimulus.^[^ ^17^ ^]^ Moreover, their dynamic nature provides a unique strategy to long term retention, as adaptation to the continuous mechanical strains within the articulating joint, prevents fragmenting, supporting extended drug retention and release kinetics (weeks to months),^[^ ^17^, ^18^ ^]^ consistent dosing and local lubrication.^[^ ^19^ ^]^ To this end, as functional hydrogels, they possess the mechanical tunability, biocompatibility, and biodegradability expected from conventional quiescent gels, making them ideally placed to overcome the limitations of in situ gelation.^[^ ^20^ ^]^

In this study, we aimed to develop an injectable steroid gel formulation that would adapt to the mechanical strain of the articulating joint, possessing an extended controlled glucocorticoid release profile to more effectively suppress markers of disease activity and joint destruction without causing off‐target deleterious side effects in tissues such as cartilage and muscle. To achieve this, gellan sheared hydrogels (GSHs) were utilized in combination with the therapeutic steroids’ cortisol and prednisolone, and their cell compatibility and capacity to suppress inflammation in both synoviocyte culture and in vivo models of arthritis assessed.

## 2 Results

### 2.1 Steroid Loaded GSHs are Injectable, Displaying Non‐Newtonian Behaviors

**Figure 1:**
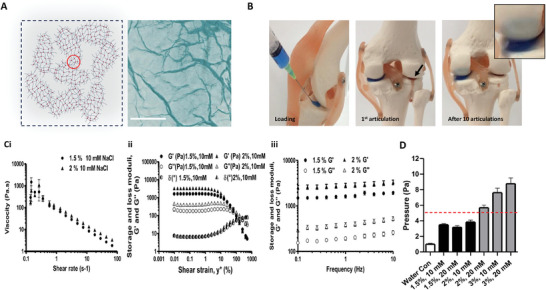
A) Schematic of known gellan sheared hydrogel structure supported by representative microscopy image of polymer strands after separation in polyethylene glycol (scale bar 50 µm). B) Representative images of blue dye labeled gellan gum sheared hydrogel (GSH) (1.5% (w/v), 10 mM NaCl; Left) and red dye labeled saline (right) following administration using a 30‐gauge needle to a plastic model of the femur‐tibia articular space and over ten 90 ° mechanical articulations captured using a high‐speed camera. Black arrows denote saline egress from joint. ci‐iii) Viscosity test, Amplitude sweep test, and Frequency sweep test of GSH formulations (1,5, and 2% gellan at 10 mM NaCl, of 1,5 and 2% gellan at 10 mM NaCl) determined by linear rheology. D) Injection pressure (Pa) required to eject GSH formulations (1,5% gellan with 10 mM and 20 mM NaCl, 2% gellan with 10 mM and 20 mM NaCl and 3% gellan with 10 mM and 20 mM NaCl) through a 30‐gauge needle relative to distilled water. Data presented as mean ± SEM of at least 3 replicates per gel formulation.

Gellan sheared hydrogels were prepared as previously reported for polysaccharide‐based fluid gels resulting in a complex microstructure formed of micro polymer networks linked by junction zones (red dots) where close packing facilitates interconnections to form a single weak continuous network (**Figure** **1**A).^[^ ^17^, ^19^, ^21^ ^]^ These were visualized under microscopy using poly(ethylene glycol) to alter the refractive index of the continuous phase. Shear separation during gelation forced the formation of quasi‐gelled interfaces (highlighted within the red circle) facilitating partial entanglements akin to a “Velcro” effect.^[^ ^22^ ^]^ Consequently, systems demonstrated a range of mechanical behaviors that allow for ease of injection and retention within articulated surfaces (Figure 1B). Further investigation into their rheological behaviors highlighted non‐Newtonian (shear thinning) flow profiles (Figure 1I). Of interest, the formulation had little effect on this behavior. Such observations were not so under small deformation, with systems showing viscoelastic responses as a function of the polymer/crosslinker concentration (Figure 1Cii‐iii).

Amplitude measurements under increasing strain ($\dot{\gamma }$) revealed solid‐like behaviors, storage modulus (G’) > loss modulus (G’’), until a critical strain in which the moduli reversed (Figure 1Cii). This behavior was characterized by polymer concentration dependency (1.5% gel: G’; ca. 1.3‐2 kPa. G’’; ca. 0.10‐0.44 kPa. – 2% gel: G’; ca. 2.2‐3.3 kPa. G’’; ca. 0.30‐0.6 kPa) with similar linear viscoelastic regions (LVRs) and resulting decay curves. The eventual crossover of the storage/loss moduli resulted in a transition from elastic to viscously dominated systems, characterized through phase angle data. The smooth reduction in storage modulus at a rate higher than that of the loss suggests flow of the material as opposed to sudden fracture, inferring liquid‐like behavior at higher strains. Further evaluation of the microstructure via controlling the frequency within the linear viscoelastic region for both the 1.5 and 2% gellan sheared hydrogel (10 mm NaCl) formulations, highlighted frequency dependent natures to both formulations, typical of sheared gel materials (Figure 1Ciii).

Translation of mechanical analysis into a more relevant patient/clinical setting was examined using glucocorticoid loaded (50 µg mL^−1^) GSHs (systematically controlling crosslinking concentration) within a 30‐gauge needle for intra‐articular delivery and quantifying required pressures to facilitate extrusion (Figure 1D). Whilst all formulations in the ranges of 1.5‐3% (w/v) polymer and 10–20 mm NaCl, could be extruded, increasing gellan % (w/v) and linker concentration resulted in an overall increase in pressure required to eject the material. At and above 3% (w/v) gellan (at 10–20 NaCl) and in 2% (w/v) GSHs above 20 mm NaCl linker, a force of greater than >5 Pa were required to extrude the gels – thus continued studies with these gels was discontinued due to being incompatible for intra‐articular injection. Wider examination of formulations with linker concentrations >40 mm (for all gellan concentrations examined), and gellan % (w/v) of >3 (10 mm NaCl) were also discontinued as a result of failing to form uniform extrusion profiles; with evidence of heterogenous gelation and increased material stiffness (Summarized in **Table** **1**).

**Table 1:** Overview of gel formulations for the application of steroid delivery in vivo. NA = not applicable.

Gellan [%] Linker (NaCl mM)	Gelation	Stable in culture (day 21)	Injectable < 5 Pa	Steroid Release	Suitable rheology for in vivo *delivery*
1.5% 10mM	Uniform	Yes	Yes	17 days	Suitable
1.5% 20mM	Uniform	Yes	Yes	17 days	Suitable
1.5% 40mM	Non‐uniform	NA	No	NA	NA
2% 10mM	Uniform	Yes	Yes	17 days	Suitable
2% 20mM	Uniform	Yes	No	NA	Suitable
2% 40mM	Non‐uniform	NA	No	NA	NA
3% 10mM	Uniform	Yes	No	17 days	Suitable
3% 20mM	Non‐uniform	NA	No	NA	NA
4% 10mM	Non‐uniform	NA	No	NA	NA

### 2.2 GSHs Display an Extended Steroid Release Profile

**Figure 2:**
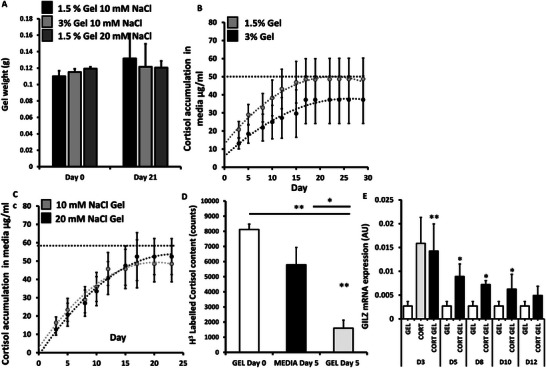
A) Gel weights (1.5‐3% gellan (w/v), 10–20 mM NaCl, 50 µg mL^−1^ cortisol) after being maintained in RPMI culture media at 0 and 21 days. B) Accumulation of cortisol into media determined by ELISA after incubation with gellan sheared hydrogel (GSH) formulations (1.5‐3% gellan (w/v), 10 mM NaCl, 50 µg mL^−1^ cortisol) over 30 days. C) Accumulation of cortisol into media from gels determined by ELISA after incubation with GSH formulations (1.5% gellan (w/v), 10–20 mM NaCl, 5 µg/ml cortisol) over 24 days. D) H3‐labelled cortisol detection in culture media and within GHS (1.5% (w/v) gellan, 10 mM NaCl) at day 5 relative to matched GSH at day 0. E) mRNA expression (AU) of Gilz in human primary macrophage cultures treated for 24 h with conditioned media collected from cortisol loaded GSH (1.5% (w/v) gellan, 10 mM NaCl, cortisol 5 µM) at days 3, 5, 8, 10, and 12, determined by quantitative RT‐PCR. Data are presented as mean ± SEM of at least 3 replicates per gel formulation. Statistical significance was determined using one‐way ANOVA with Tukey's multiple comparisons test (* *p* ≤ 0.05, **p ≤ 0.01, *** *p* ≤ 0.001).

Total gel mass was examined in culture for a period of 21 days, to study the effects of swelling and/or dispersion over time. Following the application of 100 mg aliquots of gel sample into 1 mL of culture medium (RPMI, 10% fetal calf serum) gels were incubated, with masses obtained at day 21 – following media removal (**Figure** **2A**). Gels remained clearly visible at day 21, with no significant changes in mass observed across minimum and maximum viable gellan percentages (1.5‐3.0% w/v, 50 µg mL^−1^ cortisol) at NaCl linker concentrations of 10–20 mm. Controlled delivery of the glucocorticoid was examined under typical culture conditions to determine the impact of the delivery vehicle formulation on release duration. Accumulation and relative release of the glucocorticoid cortisol within the surrounding medium over a period of 30 days were assessed across minimum and maximum viable gellan percentages (1.5‐3.0% w/v) and NaCl linker concentrations (10‐20 mm) in gels loaded with 50 µg mL^−1^ cortisol (Figure 2B,C).

Comparison of release plots for systems at constant crosslinker concentration (10 mm) revealed that cortisol accumulation, for all systems studied, plateaued at day 17 reflecting the total steroid content loaded within the gel (Figure 2B). Moreover, release profiles closely fitted (R^2^ > 0.98) semi‐empirical models for hydrogel systems (power model: *f* = *Kt ^n^* where f is the amount of drug released; K, is the release velocity constant; t, is the time and; n, is the exponent of release)^[^ ^23^ ^]^ Both 1.5% and 3.0% gel formulations exhibited release exponents suggestive of typical Fickian diffusion (n = 0.49 and 0.50, respectively). Whilst steroid release showed a trend toward decreased release in the 3.0% gellan formulation, differences between both groups were not significant.

To assess the impact of NaCl linker concentration on steroid release properties, both accumulation of cortisol within the surrounding medium and relative release from the gel over a period of 25 days were assessed across minimum and maximum viable NaCl linker concentrations (10 versus 20 mm) at 1.5% w/v gellan (Figure 2C,D). No differences in the rate of cortisol accumulation within the media were evident between gels at 10 and 20 mm of NaCl linker, however, although still modeled via as a power function (R^2^ > 0.95) the release exponent increased (n = 0.66 and 0.8, respectively) suggesting a shift toward kinetics more closely described as zero‐order (Figure 2C). To better understand retention of the active within the gel, tritium labelled cortisol gels were generated and studied over a period of 5 days (Figure 2D). This revealed that 21.6 ± 5.8% of the total steroid was retained within the gel at day 5, whilst 78.3 ± 14.9% of the steroid had been released into the surrounding media. Media collected from cortisol loaded 1.5% gellan sheared hydrogels (10 mm NaCl linker) and vehicle loaded counterparts were collected for functional analysis of glucocorticoid action, using *glucocorticoid‐induced leucine zipper* (*GILZ*) mRNA expression (AU) in primary human macrophages (Figure 2E). At day 3, media collected from cortisol loaded GSHs resulted in a marked upregulation of *GILZ* expression (day 3; 5.2‐fold; *p* < 0.01) relative to those treated with media collected from vehicle loaded control GSHs. This induction in *GILZ* expression diminished gradually over time (day 5; 3.25‐fold, *P* < 0.05, day 8; 2.6‐fold, *p* < 0.05, day 10; 2.3‐fold, *p* < 0.05, day 12; 1.8‐fold, NS) up to day 12 where a significant upregulation was no longer be detected.

### 2.3 Steroid Loaded GSHs Suppress Inflammatory Macrophage and Osteoclast Activation

**Figure 3:**
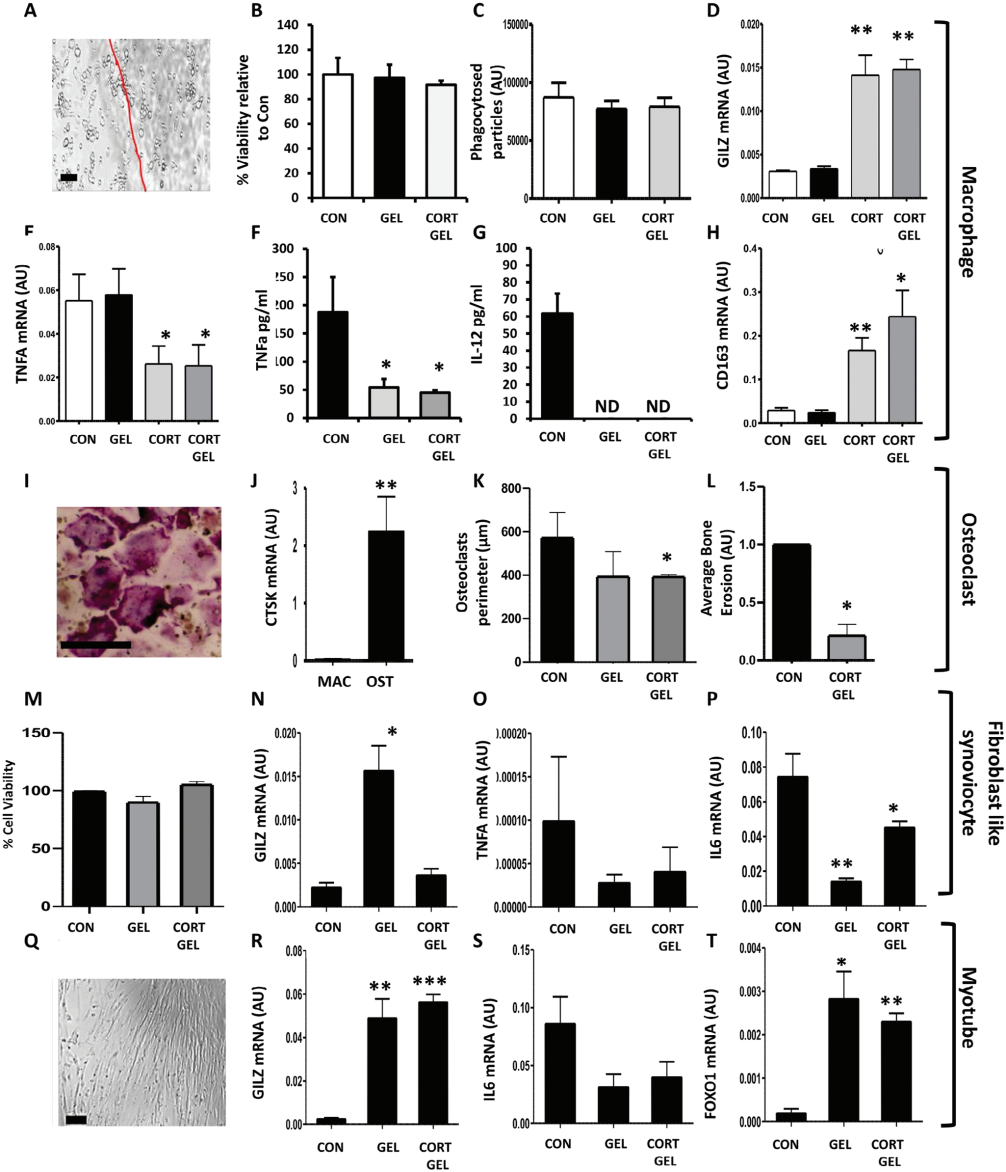
All groups consisted of treatments with either 25 µL of gellan sheared hydrogel (GSH) (1.5% gellan (w/v), 10 mM NaCl,) containing either vehicle (Gel), 5 µM cortisol (Cort Gel) relative to untreated control for 48 h for functional analysis or 24 h for mRNA analysis. A) Representative image by microscopy (scale 50 µl), B) cell viability determined by MTT, and C) phagocytosis determined by Vybrant™ E. coli phagocytosis assay in primary human macrophages. D‐F) mRNA expression (AU) of GILZ, TNFA, and CD163 determined by quantitative RT‐PCR and G‐H) cytokine release of TNFα and IL‐12 determined by ELISA in primary macrophages. I) Representative image of TRAP‐stained primary osteoclasts by microscopy (scale 50 µl). J) mRNA expression (AU) of CTSK determined by quantitative RT‐PCR, K) Primary human osteoclast perimeter (µM), and L) Average Bone erosion scores (AU) in primary human osteoclasts. M) Cell viability determined by MTT and N‐P) mRNA expression (AU) of GILZ, TNFA, and CD163 determined by quantitative RT‐PCR in primary human fibroblast‐like synoviocyte culture. Q) Representative image by microscopy (scale 50 µL) of primary human myotube culture. R‐T) mRNA expression (AU) of GILZ, IL6, and FOXO1 determined by quantitative RT‐PCR in primary human myotubes. Data are presented as mean ± SEM of at least three primary cultures from three independent patient donors. Statistical significance was determined using one‐way ANOVA with Tukey's multiple comparisons test (* *p* ≤ 0.05, **p ≤ 0.01, *** *p* ≤ 0.001).

To examine cell compatibility, immunomodulatory and anti‐inflammatory properties of GSHs loaded with cortisol, injectable formulations made with 1.5% gellan, 10 mm NaCl and containing 500 µg mL^−1^ cortisol were generated and maintained with cultures of human primary cultures of macrophages, synovial fibroblasts, bone resorbing osteoclasts, and muscle cells (**Figure** **3**). Under 20x magnification, inflammatory activated macrophages were evident adjacent to and below the sheared hydrogel (Figure 3A). Examination of viability by 3‐(4,5‐dimethylthiazol‐2‐yl)‐2,5‐diphenyltetrazolium bromide (MTT) assay and measures of cell cytotoxicity in macrophages showed no significant reduction at 48 hours with vehicle and cortisol loaded GSHs relative to untreated controls (Figure 3B; Figure S1A, Supporting Information). The acutely induced glucocorticoid responsive gene *GILZ* was measured in primary macrophages at 24 h, following culture with either vehicle or cortisol loaded GSHs, relative to cortisol or untreated controls (Figure 3D). Culture with cortisol and cortisol loaded gels resulted in a marked upregulation of *GILZ* expression (4.6‐fold; *p* < 0.01, 4.9‐fold; *p* < 0.01) relative to untreated controls, whilst vehicle loaded gel did not significantly influence gene expression (Figure 3D). Analysis of the potent pro‐inflammatory mediator *tumor necrosis factor alpha* (*TNFα*) revealed that both direct exposure to cortisol and cortisol loaded gels significantly suppressed mRNA expression (2.19‐fold; *p* < 0.05, and 2.27‐fold; *p* < 0.05) and secretion (Gel only; 187.3 ± 58.2 pg mL^−1^ versus Cort; 54.31 ± 8.8, *p* < 0.05, and Cort Gel; 44.7 ± 3.9, *p* < 0.05) relative to vehicle loaded formulations (Figure 3E,F). Similar results were evident in secretion interleukin 12 (IL‐12), with both cortisol and cortisol loaded GSHs reducing secretion to a level below the limit of detection (Figure 3G). In contrast, mRNA expression of the pro‐resolving macrophage scavenger receptor *cluster of differentiation 163* (*CD163)* was significantly upregulated in these groups relative to vehicle loaded gels (6.9‐fold; *p* < 0.01, 9.91‐fold; *p* < 0.05) (Figure 3H). Multinucleated TRAP+, CTSK+ osteoclasts were generated from human macrophages after 48 hours incubation with macrophage colony stimulating factor (M‐CSF) and receptor activator of nuclear factor kappa beta (RANKL) (Figure 3I,J). Cortisol loaded GSHs effectively suppressed osteoclast perimeter (Con Gel; 572.3 ± 118.7 µm versus Cort Gel; 391.5 ± 115.4; *p* < 0.05) and bone chip erosion scores (79.3%; *p* < 0.05) relative to vehicle loaded counterparts (Figure 3K,L). Mitochondrial function as a measure of viability and cytotoxicity were examined in primary fibroblast like synoviocytes following incubation for 48 h with either vehicle or cortisol loaded GSHs (Figure 3M; Figure S1B, Supporting Information). No changes were evident between these groups relative to untreated controls. In the same synoviocytes, GILZ, *TNFα*, and *interleukin 6* (*IL6*) mRNA expression were measured to determine immunoregulation by these formulations. Cortisol induced *GILZ* and suppressed *IL6* expression relative to controls, whilst cortisol loaded GSHs resulted in a partial suppression of *IL6* only (Figure 3N–P). Neither intervention influenced *TNFα* expression. Lastly, cortisol GSHs and direct cortisol treatments were examined relative to vehicle loaded GSHs in human primary myotube cultures (Figure 3Q–T). After differentiation, myotubes formed clearly defined fibers under 10x magnification (Figure 3Q). Both cortisol and cortisol gel formulations potently upregulated the anti‐inflammatory glucocorticoid response gene *GILZ* relative to vehicle loaded GSHs (20.3‐fold; *p* < 0.01, 23.3‐fold; *p* < 0.001) and a trend toward decreased *IL6* expression (2.7‐fold & 2.2‐fold; NS) (Figure 3R,S). Conversely, catabolic *forkhead box protein O1* (*FOXO1*) expression was markedly upregulated in both cortisol and cortisol gel formulations (14.7‐fold; *p* < 0.01, 12.1‐fold; *p* < 0.05) relative to vehicle control (Figure 3T). Together these data indicate that cortisol loaded gellan sheared hydrogels suppress inflammatory cytokine output in macrophage and fibroblast like synoviocyte populations, whilst suppressing osteoclastic bone resorption.

### 2.4 Intra‐Articular Delivery of Steroid Loaded Sheared Hydrogels Suppress Joint Destruction

**Figure 4:**
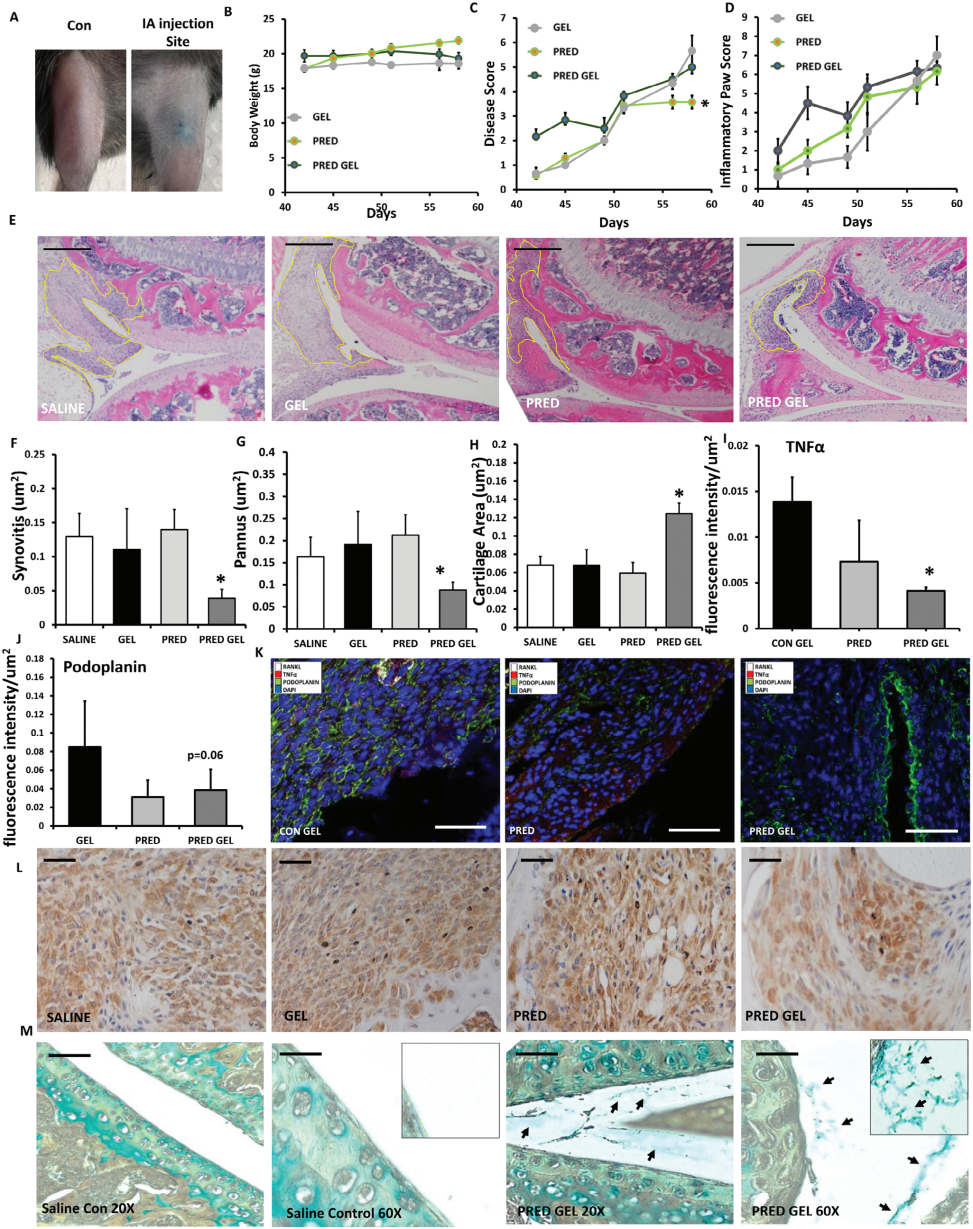
All groups consisted of intra‐articular injection with 10 µL of either saline control (CON), blank GSH (GEL: 488 1.5% gellan (w/v), 10 mM NaCl), methylprednisolone sodium succinate (PRED: 10 mg mL^−1^) or GSH loaded with methylprednisolone sodium succinate (PRED GEL: Gel: 1.5% gellan (w/v), 10 mM NaCl, Pred 10 mg mL^−1^). A) Representative image of Coomassie blue dye loaded gellan sheared hydrogel (GSH) administered into the intra‐articular space in the TNFtg murine model of polyarthritis. B) Body weight, C) disease score, and D) Inflammatory paw score D) measured over 21 days in the TNFtg model of polyarthritis following intra‐articular injection. E) Representative sagittal images of formalin‐fixed paraffin embedded knee joints, stained with hematoxylin and eosin, and F) measurement of synovitis, G) sub‐chondral pannus formation, and H) surface cartilage area in TNFtg animals at day 21 following treatment. I‐K) Fluorescence intensity/um^2^ of synovial tissue as a measure of expression of TNFα and podoplanin, and representative images of fluorescent staining for RANKL, TNFa, podoplanin, and DAPI, determined by confocal immunofluorescence area in TNFtg animals at day 21 following treatment. L) Representative GILZ staining within synoviocytes was determined in the same groups within articular synovium by immunohistochemistry. M) Representative alcian blue staining within the articular space was performed in TNFtg animals after 21 days following injection with either saline control (Con) or GSH loaded with methylprednisolone sodium succinate at 20 and 60X magnification. Black arrows denote positively stained GSHs. Data are presented as mean ± SEM of at least three six animals per group. Statistical significance was determined using one‐way ANOVA with Tukey's multiple comparisons test (* *p* ≤ 0.05, **p ≤ 0.01, *** *p* ≤ 0.001). (scale bars, 50 µm).

The transgenic(TNF)197Gkl (TNFtg) murine model of polyarthritis was utilized to assess the efficacy of steroid loaded GSHs in vivo. Due to the reduced biological efficacy of the glucocorticoid cortisol in mouse models, formulations containing the therapeutic steroid methylprednisolone sodium succinate were utilized. Formulations containing methylprednisolone sodium succinate possessed identical linear and nonlinear rheological properties, behaving as an injectable non‐Newtonian fluid. Animals were split into three wings receiving intra‐articular injections of either 10 µL of methylprednisolone sodium succinate loaded GSH (10 mg mL), an equivalent dose of methylprednisolone sodium succinate in saline (standard of care) or vehicle loaded GSH into the right knee. All animals received a matched control saline injection into the left knee. Injection into the intra‐articular space were delivered medial to the patellar tendon, with GSHs containing Coomassie blue dye to visualize delivery into the joint (**Figure** **4A**). Total body weight between animals receiving methylprednisolone sodium succinate, vehicle loaded gel formulations, and methylprednisolone sodium succinate counterparts remained stable across all groups between days 42 to 58 (Figure 4B). Examination of systemic measures of disease activity, factoring mobility, social behavior, pain behavior, and joint inflammation revealed no significant differences between vehicle and methylprednisolone sodium succinate loaded gel formulations, whilst methylprednisolone sodium succinate alone resulted in a significant decrease at day 58 (Pred Gel; 5.0 ± 2.1 versus Pred; 3.57 ± 0.8. *p* < 0.05) (Figure 4C). In contrast, no change was apparent in direct local joint inflammation scores between groups (Figure 4D). To examine local joint destruction within the intra‐articular space, joint histology was examined following day 58 to quantify synovitis, invasive pannus formation and cartilage erosion (as previously reported)^[^ ^24^ ^]^ Synovitis did not change in joints from animals receiving methylprednisolone sodium succinate or the saline vehicle loaded gel formulation relative to matched saline injected joints, whilst a significant reduction was apparent in those receiving the prednisolone GSH (Saline Con; 0.129 ± 0.032 versus Pred Gel; 0.039 ± 0.017 um^2^. *P* < 0.05) (Figure 4E,F). An identical pattern was observed with pannus invasion in subchondral bone, with a significant decrease only evident in the joints of animals receiving methylprednisolone sodium succinate loaded GSH injection (Saline Con; 0.163 ± 0.051 versus Pred Gel; 0.088 ± 0.018 um^2^. *P* < 0.05) (Figure 4E,G). Conversely, cartilage area was increased in joints treated with methylprednisolone sodium succinate loaded GSHs relative to saline injected controls (Saline Con; 0.068 ± 0.0042 versus Pred Gel; 0.0124 ± 0.0064 um^2^. *P* < 0.05) (Figure 4E,H). Several markers of synovial inflammation, including receptor activator of nuclear factor kappa beta (RANKL), TNFa, and podoplanin were next examined in the methylprednisolone and methylprednisolone GSH treated animals relative to animals receiving GSH only (Figure 4I–K). A significant reduction in TNFα expression was apparent in animals receiving methylprednisolone GSH relative to GSH treated controls (*P* < 0.05), whilst only a trend toward reduced expression was evident in methylprednisolone treated group (Figure 4I,K). Similarly, a trend toward reduced podoplanin expression was evident in both groups receiving methylprednisolone and methylprednisolone GSH (approaching significant in the latter; p = 0.06) (Figure 4j,K). This was characterized by a reduction in staining within the sublining in the methylprednisolone and methylprednisolone GSH treated groups. In contrast, RANKL was only weakly expressed within the synovium and showed no significant change in relative expression. To examine the induction of glucocorticoid signaling within the joint, *Gilz* was examined by immunohistochemistry within the inflamed synovium of injected joint and sub chondral bone marrow (Figure 4L). Substantial basal *Gilz* expression was apparent within Saline injected control joints throughout the synoviocyte populations of the inflamed synovium, and more selectively within lining cells, osteoclasts and macrophage‐like cells of the sub‐chondral bone marrow. Similar patterns were seen across all treatment groups, with no evidence to suggest altered glucocorticoid induced *Gilz* expression between treatment wings at the final time point examined. To image the steroid loaded GSHs within the articular space, alcian blue staining was used to highlight the gel polysaccharide as previously reported (Figure 4M)^[^ ^25^ ^]^ These revealed a mixed pattern of gellan fragments within the articular space, rather than a uniform homogenous distribution, that was entirely absent in saline injected controls. These appeared independent from local resident and immune cells infiltrates at day 21. Collectively, these data indicate that methylprednisolone sodium succinate loaded gel formulations are effective at suppressing synovitis and joint destruction at day 21 following intra‐articular injection.

### 2.5 Steroid Loaded GSHs do not Cause Juxta Articular Muscle Wasting

To examine off‐target actions of intra‐articular delivery of steroid loaded GSH interventions, local quadricep muscle was examined at the site immediately adjacent to injected joints. Wet muscle weights did not significantly differ relative to the matched saline controls (saline con, 81 + mg; Pred, 83 + mg; Gel, 81 + mg, Pred Gel; 80 + mg. NS) (Figure S2A, Supporting Information). Analysis of quadriceps fiber size supported these findings, with no changes in average fiber size across the prednisone, prednisone loaded gel, and vehicle loaded gel intervention wings relative to saline controls (Figure S2B‐E, Supporting Information). Together, these data suggest that neither the standard of care methylprednisolone sodium succinate injection, or the vehicle and methylprednisolone sodium succinate gel interventions were sufficient to drive localized muscle wasting when delivered by intra‐articular injection at this time point.

## 3 Discussion

Classical intra‐articular glucocorticoid formulations take the format of sodium succinate salts of steroids (e.g., methylprednisolone acetate, dexamethasone, cortisol, betamethasone, prednisolone, and triamcinolone), increasing solubility and facilitating delivery at high doses within saline; compensating for their rapid clearance from joints.^[^ ^26^ ^]^ In this study, we investigated the suitability of gellan sheared hydrogels (GSHs) as a vehicle for slow‐release glucocorticoids drug delivery by intra‐articular joint injection. These hydrogels, characterised by their non‐Newtonian fluid properties, were suitable for injection via a 30‐gauge needle under shear stress, yet exhibited solid‐like gel properties at rest. Consequently, these formulations were able to transition within the joint between fluid and solid‐like properties, adjusting to the mechanical articulation and coating the chondral surface. When loaded with the anti‐inflammatory agents, these formulations demonstrated extended‐release profiles, in vitro, lasting up to 17 days. Release profiles highlighted typical Fickian diffusion as previously reported in polymer gels, suggesting a dependence on the gradient in/out of the delivery vehicle.^[^ ^27^ ^]^ As such, release kinetics were dependent on the formulation of the delivery vehicle but governed by crosslinking concentration (shifting release away from 1st toward 0 order with increasing concentration) and not gellan content. These data would indicate that surrounding aqueous phase volume is a central feature influencing the steroid release kinetics of these gels. However, this aspect of release would be markedly altered in vivo, where aqueous synovial fluid volumes are significantly less (relative to gel), and the gel would be exposed to intermittent mechanical disruption. Consequently, Fickian diffusion may play less of a role in the release kinetics within the joint, whilst mechanical disruption of the gel under articulation (in vivo) may be a more dominant factor influencing steroid release properties as the gel transitions between solid‐liquid‐solid. This may contribute to the extended efficacy of the steroid loaded gels used in this study when used in vivo relative to in vitro. To date, no purpose designed models exists for measuring drug release from a material in a closed, low aqueous environment exposed to mechanical manipulation that would address this.

**Figure 5:**
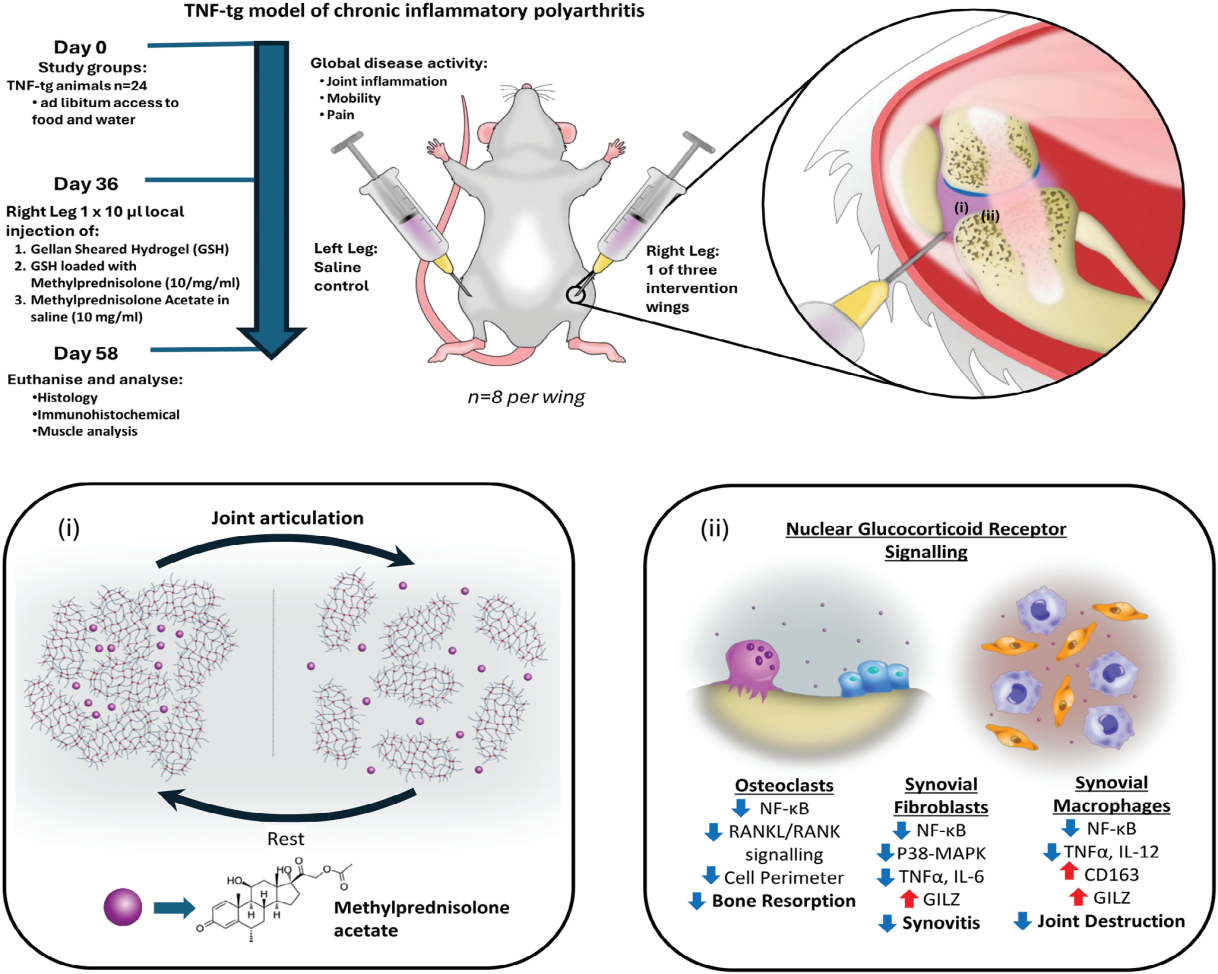
Schematic demonstrating the in vivo study design. TNF‐tg animals were maintained for 36 days from birth up to onset of early joint inflammation before receiving one of three therapeutic intervention wings by intra‐articular injection into the right leg, consisting of either gellan sheared hydrogel loaded with saline, gellan sheared hydrogel loaded with methylprednisolone sodium succinate (10 mg mL^−1^), or methylprednisolone sodium succinate made up in saline. All animals received a saline control injection into the left leg for the purpose of providing a matched control. Animals were scored for systemic and local disease activity up to day 58 when animal were euthanized and tissues harvested for analysis. (ii), schematic showing the known polymer structure of gellan sheared hydrogel and the predicted location of methylprednisolone both at rest in the resting joint and under mechanical articulation. (ii), schematic detailing the known changes in local inflammatory signaling mediated by glucocorticoid loaded hydrogels in primary cell cultures of joint cells, including osteoclasts, synovial macrophages, and synovial fibroblasts.

Irrespective of the formulation all systems were found to be cell compatible, supporting prior examination of their in vitro and in vivo safety profiles.^[^ ^28^, ^29^, ^30^ ^]^ In vivo, GSHs loaded with the widely used therapeutic steroid methylprednisolone sodium succinate effectively suppressed local joint destruction and synovitis without driving local muscle wasting at the site of injection (**Figure** **5**). Histological analysis revealed that these glucocorticoid loaded formulations attenuated local joint destruction, reduced synovitis and pannus formation, and preserved articular cartilage. Thus, these formulations hold promise as an effective means of delivering therapeutic steroids via intra‐articular injection for joint diseases such as rheumatoid and osteoarthritis, offering prolonged relief with reduced off‐target side effects.

Several prior studies have examined the therapeutic applications of hydrogels such as gellan, agarose, gelatin, hyaluronic acid, and acrylamide to deliver therapeutics to the intra‐articular space, with several exploring their capacity to promote cartilage regeneration.^[^ ^31^, ^32^, ^33^, ^34^ ^]^ To overcome the mechanical hurdles of delivery after gelation, these formulations have utilized in situ gelation strategies. These have included temperature dependent sol‐to‐gel transition with negative thermosensitive hydrogels, and gelation induced by physiological conditions of pH or ionic composition.^[^ ^13^, ^35^ ^]^ These strategies create unique challenges for therapeutic handling and delivery in a clinical setting and have yet to prove clinically viable. Further unique approaches have included hydrogel synthesized using a Schiff‐base reactions to crosslink polymers and create injectable hydrogels suitable for glucocorticoid loading, and that preserving cartilage in a model of osteoarthritis.^[^ ^36^ ^]^ However, shear thinning materials, with rapid recovery of elastic properties represent a class of material that can be readily synthesized and tuned to favor a myriad of applications have proven to overcome many of the practical problems associated with minimally invasive injectable hydrogel delivery. To this end, the GSH formulations examined in this study possessed ideal shear thinning properties required for intra‐articular injection in clinical practice.

As a well‐defined factor that influences the gel polymer network, NaCl linker and gellan (w/v) were manipulated in this study to examine their influence on steroid release properties of GSHs.^[^ ^17^ ^]^ Between 1.5‐3% gellan and 10–20 mM NaCl, a comparable profile of steroid release was observed, characterized by an initial rapid Fickian diffusion from the gel over the first 48 h and followed by a more gradual release over 17 days. While this change appeared to be independent of the gel content or linker concentration within the viable ranges explored, a trend toward a reduced rate of diffusion appeared to result from increasing the gellan % (w/v). These findings would suggest that the majority of the steroid largely occupies the interstitial aqueous phase of the gel upon synthesis, where it would be free to diffuse out through the polymer chains of the matrix into the surrounding medium.^[^ ^37^ ^]^ The more gradual release of remaining drug would then be dependent on interactions between the steroid and the gel fragments, as has previously been reported in microsphere‐microcrystal‐gel delivery and hyaluronic acid hydrogels gels.^[^ ^35^, ^38^ ^]^ Future approaches aiming to further extend the steroid release profile might explore the combination of GSHs with alternative slow‐release steroid formulations, such as poly (lactic‐co‐glycolic acid) (PLGA) microspheres, polymeric micelles, or PEGylated liposomal packaging.^[^ ^39^, ^40^, ^41^ ^]^ Many of the therapeutic glucocorticoid variants, including triamcinolone sodium succinate, triamcinolone hexacetonide, betamethasone sodium succinate and betamethasone sodium phosphate already exhibit improved solubility and chemical properties that would be predicted to further enhance steroid release kinetics when combined with gellan sheared hydrogels.^[^ ^26^ ^]^ In particular, the steroid sodium succinate salts may be utilized as an alternative crosslinker to NaCl to tailor hydrophobicity and drug retention. Notably, it remains unclear how the release kinetics of steroid loaded GSHs vary between in vitro and in vivo models. The continual mechanical strain In vivo and reduced surrounding fluid volumes (absent from in vitro release modelling) within the self‐contained joint capsule may enhance or slow in vivo release. In this study, we attempted to retrieve surrogate measures of glucocorticoid release (*Gilz* signaling) to examine drug release at these end stage timepoints. However, these findings were inconclusive, and further analysis of steroid release at earlier time points is now required to assess whether the steroid slow‐release properties of these GSH formulations were contributing to the improved outcomes in these animals.

Given that the synovial fluid is continually reabsorbed into the circulation from the joint, it is unsurprising that traditional injectable steroid formulations are rapidly detected within the circulation following intra‐articular administration, peaking at 2–12 h post injection.^[^ ^4^ ^]^ To mitigate the impact of steroid loss from the joint and consequent loss of local exposure following delivery, these formulations are administered at relatively high doses to maximize the anti‐inflammatory and immunomodulatory action for the duration in situ. This feature underpins the rapid action, but short duration of these interventions, and contributes to the dose dependent deleterious side effects of these steroids seen in local cartilage and bone.^[^ ^42^ ^]^ In this study the standard of care with methylprednisolone sodium succinate injection alone, resulted in a suppression in global disease activity scores without improving local joint destruction. This may reflect a similar phenomenon as seen in patients, with initial increased systemic exposure to the steroid in this group as the glucocorticoid is lost from the joint capsule.^[^ ^4^, ^43^ ^]^ In contrast, the extended and more controlled release of steroid from GSHs may have the advantage of reducing the initial dose, and frequency of follow up injections needed to control inflammation. Given that the high doses of traditional steroids directly drive processes of apoptosis in bone forming osteoblasts and cartilage producing chondrocytes, this feature of GSHs now requires further investigation.^[^ ^42^, ^44^, ^45^ ^]^ Certainly, given the rapid early egress of steroids in injectable hydrogel formulations, future refinement approaches might focus on reducing this initial loss, to favor extended drug release further still.^[^ ^33^, ^35^, ^38^ ^]^

Several studies have explored whether intra‐articular gels improve articulation within the joint through the promotion of chondrogenesis and recovery of cartilage.^[^ ^32^, ^35^, ^46^ ^]^ These have utilized gellan and hyaluronic acid hydrogels in combination with glucocorticoids to provide a chondrogenic scaffold that promotes cartilage repair. Independently of glucocorticoids, hydrogels have been predicted to facilitate cartilage regeneration by promoting cell migration and chondrocyte differentiation.^[^ ^47^ ^]^ In this study, there was little evidence that the gellan sheared hydrogels alone promoted cartilage repair. However, a marked increase in articular cartilage was evident in joints injected with methylprednisolone sodium succinate loaded GSHs. This suggests that the ability of glucocorticoids to suppress the induction of pro‐inflammatory mediators and matrix metalloproteinases that drive cartilage erosion is the more likely driver of cartilage preservation in this setting, supporting findings from similar studies.^[^ ^35^, ^36^, ^48^ ^]^

In regards to clearance of gellan from the joint, several studies report that gellan hydrogels activate and promote macrophage phagocytosis mediated removal in vivo.^[^ ^49^ ^]^ In this study, in vivo histology imaging of gels at day 21 after injection using alcian blue suggested that distinct fibers sit adjacent to the joint surface lining the chondral surfaces. These observations mirrored findings in the *ex vivo* mechanical modeling of gel distribution within artificial joints and support the concept that GSHs distribute across the joint surface under articulation. Of note, there was no significant indication of phagocytic immune cell clearance of gels either within the joint. Whilst this supports the concept that these formulations are cell and tissue compatible, it highlights that further work is needed to characterize the long‐term clearance of these materials in vivo. Further aspects of the physical properties of these gels should also be considered when applied in this context, such as their viscoelastic nature, that would be predicted to store and dissipate energy in a similar fashion as cartilage. Whilst this study did not focus on joint lubrication, further exploration of whether these formulations would reduce friction within the joint, in a similar fashion as that reported for microfluidic electrosprays, would be of significant interest in the context of disease settings such as osteoarthritis, where contact wear within the joint contribute to disease pathophysiology.^[^ ^30^, ^50^ ^]^ Both the gel percentage and linker concentration influence these properties and would merit further analysis in this regards.^[^ ^51^, ^52^ ^]^ These features may ultimately possess efficacy in suppressing pain driven by these mechanical processes.

## 4 Conclusions

In this study, we investigated the suitability of gellan sheared hydrogels (GSHs) as a vehicle for slow‐release glucocorticoids drug delivery by intra‐articular joint injection. These hydrogels were cell‐compatible and characterized by non‐Newtonian fluid properties, making them suitable for injection using narrow gauge needles. Of note, they displayed an extended steroid drug release profile, determined by Fickian diffusion through the crosslinked polymer, resulting in increased efficacy in suppressing joint destruction and synovitis in a murine model of inflammatory arthritis. Limitations to this study include a need for greater understanding of how these materials are cleared over time, and how they influence the drug release profiles of other alternative non‐steroidal and disease modifying therapeutic interventions. However, this study supports the concept that gellan sheared hydrogels hold promise as an effective means of delivering intra‐articular therapeutic steroids at lower doses and with reduced off target side in diseases such as rheumatoid and osteoarthritis. Further work is now required to understand whether they can also improve lubrication and pain in appropriate models of polyarthritis and explore their capacity to support cartilage and local tissue repair through the incorporation of further bioactive factors.

## 5 Experimental Section

### Gellan Fluid Gels

Sheared hydrogels containing low acyl gellan gum (gellan) powder percentages between 1.5‐4% (w/v), and NaCl linker concentrations between 10 and 40 mm were synthesized at a fixed mixing rate. Briefly, gellan powder (w/v) was added to MilliQ water containing 5% phosphate buffered saline (PBS) (v/v), 5% (v/v) NaCl linker and heated to 90 °C. Once fully dissolved the hydrocolloid solution was cooled to 60 °C under constant mixing (300 rpm) using a magnetic stirrer. Upon reaching a temperature of 40 °C, glucocorticoids, dissolved in either sterile water or ethanol were slowly added at 5% (v/v) during shearing to generate gels containing either cortisol (50 µg mL^−1^) or methylprednisolone sodium succinate (10 mg mL^−1^). Formulations with a material stiffness that exceeded the capacity for mechanical shearing, resulting in partial or non‐uniform gelation and were discontinued.

### Rheometric Characterization

Fluid gel mechanical properties were investigated using a rotational Kinexus Ultra+ Rheometer (NETZSCH, Germany) equipped with a serrated parallel plate geometry (1mm gap, 40 mm diameter), to reduce slip. All tests, unless stated otherwise, were conducted at 20 °C. Flow profiles were conducted under controlled shear rate using a 2 min ramp time between 0.01 and 100 s^−1^. Small deformation testing was conducted using frequency and amplitude sweeps. Amplitude sweeps were performed at 1 Hz between 0.1 and 500% strain. The linear viscoelastic region (LVR) was calculated and a strain common to all systems used to undertake frequency testing between 10 and 0.01 Hz.

### Radiolabeled Release Assay

1.5% gellan, 10 mm NaCl, 500 uM Cortisol with 2 uCi uL^−1^ of tritiated Cortisol was prepared to perform a radiolabeled corticosteroid release assay. 50 uL gel was maintained in RPMI complete media (media used for primary macrophage culture) for 5 days and the media and the gel were processed. Steroids were extracted from medium using dichloromethane (5–10 vol) and spotted onto a thin‐layer chromatography (TLC) plate. TLC plates were analyzed using a Bioscan imaging detector (Bioscan, Washington, DC, USA) and the fraction of released steroid was calculated by comparing with a control. All experiments were carried out in triplicate.

### Measuring Ease of Extrusion from a Syringe

The pressure (g m^−2^) required to extrude gel formulations from a 30‐gauge needle was assessed using a digital pressure sensor (Linton Instrumentation).

### Primary Macrophage and Osteoclast Culture

All cytokines were purchased from PeproTech, UK or Biotechne. Macrophages were generated using monocytes obtained from blood cones from healthy fully anonymized donors, from the NHS Blood and Transplant Centre, Birmingham. This was approved by the University of Birmingham Ethics Committee, under ethical agreement ERN_14‐0446. cluster of differentiation 14 (CD14)+ monocytes were isolated from blood using the RosetteSep Human Monocyte Enrichment Cocktail (Stem Cell, UK), as per manufacturer's guidelines. Monocytes were differentiated into undifferentiated (M0) unpolarised macrophages by culturing with 10% fetal bovine serum (FBS), 1% penicillin & streptomycin, and M‐CSF 20 ng mL^−1^) for 6 days before being classically activated (M1‐like) by culturing with interferon γ (IFNγ)/TNFα (10 ng mL^−1^) for 24 h. Osteoclasts were generated by supplementation of M0 macrophages with 1ng mL^−1^ RANKL and M‐CSF 20 ng mL^−1^) over 72h.

### Isolation and Culture of Human Fibroblasts

All reagents used in cell culture were obtained from Sigma (Poole, UK) unless otherwise stated. Fibroblasts were isolated from biopsies of matched skin, bone marrow, and synovium removed during total knee arthroplasty from consenting patients who fulfilled the 2010 ACR/EULAR criteria for rheumatoid arthritis following ethical approval (REC reference 14/ES/1044 & 071H12041). Fibroblasts were isolated by mechanical digestion of tissue followed by dissociation in 5 mmol L^−1^ EDTA for 2 h. Dissociated tissue was then washed and transferred to a culture flask. The fibroblasts were then cultured through to a maximum of seven passages in complete fibroblast medium consisting of RPMI‐1640, 1% (vol/vol) non‐essential amino acids, 1% penicillin/streptomycin, 1% sodium pyruvate, 2 mmol L^−1^ glutamine, and 20% heat‐inactivated fetal bovine serum (Labtech International, Sussex, UK). Fibroblasts were treated with gellan gel formulations (10 µL) for 24 h before harvesting.

### Myotube Cell Culture

Adult patients with hip osteoarthritis consented to collection of quadriceps muscle biopsies (150‐200 mg) during elective joint replacement surgery, following ethical approval (REC reference 14/ES/1044 & 071H12041). Fresh muscle tissue was coarsely minced with a scalpel prior to digestion in trypsin‐EDTA at 37 °C for 15 min. Cell suspensions were transferred into 0.2% gelatine‐coated flasks for culture of myoblasts in Ham's F10 media (N6013, Sigma), 100 µg mL^−1^ penicillin/streptomycin, 20% fetal bovine serum. Media was renewed every two days. Myoblasts were passaged at 60–70% confluence and used for experiments between passage 2 and 4. To differentiate myoblasts into myotubes, proliferation media was replaced with differentiation media (Ham's F10 media (N6013, Sigma), 100 µg mL^−1^ penicillin/streptomycin, 6% horse serum (heat inactivated, Gibco)). Cells were maintained in differentiation media for a minimum of eight days until distinct myotubes formed.

### TRAP Staining and Osteoclast Analysis

Osteoclast cultures were fixed in 4% formalin in phosphate buffered saline (PBS). Briefly, cells were washed twice in PBS before incubation for 40 min at 37 °C in tartrate‐resistant acid phosphatase (TRAP)staining solution consisting of sodium acetate anhydrous 1 M, L+ tartaric acid 1 M, glacial acetic acid 3%, fast red violet LB salt (0.5g mL^−1^) and naphthol AS‐MX phosphate substrate Mix (0.5 mL per 100 mL) at pH 4.7‐5.0. Cells were then washed twice with in PBS. Area, perimeter and number of osteoclasts were analyzed using Image J. Light microscopy at 10x magnification across 5×500 µm^2^ fields of view.

### In Vitro Bone Resorption Assay

After differentiation, osteoclast cultures were trypsinized and seeded directly onto bovine dentine chips to evaluate in‐vitro osteoclast functionality, osteoclasts were seeded directly onto dentine chips (BoneSlices.com, Odense, Denmark) on a 96 well plate at 200 00 cells per well. Osteoclasts were incubated on dentine chips, in the presence of either TNFα (10 ng mL^−1^), cortisone (1000 nmol L^−1^) or both for 48 h at 37 °C. Bone chips were then washed in PBS and gently scraped to remove osteoclasts using a cotton bud. Bone erosions were visualized following staining with chips were 1% toluidine blue solution for 15 s, followed by washing in 70% Ethanol. Bone chips were individually scored across the surface using light microscopy (20x magnification). Bone erosions were scored within 3 categories (Small Pits <20 µm = 1, medium trenches <50 µm = 2 and large trenches >50 µm = 3). Scores from at least three chips per intervention, across 4 independent donors were assessed.

### Cell MTT and Cytotoxicity Assay

Colorimetric MTT assay was utilized as a measure of cell viability and was performed as per the manufacturer's instruction (Merck, UK, CT02). Briefly, cells were incubated with formazan substrate for 4 hours at 37 °C after exposure to gel formulations for 48 h. Following subsequent addition of DMSO absorbance was read at 570 nm (reference wavelength 630 nm) using a spectrophotometer. Cytotoxicity was determined following trypan blue staining after exposure to gel formulations for 48 h. Cells were counted under a hemocytometer and percentage dead cells determined relative to total cell count.

### Phagocytosis Assay

Phagocytosis was assessed with the Vybrant *E. coli* Phagocytosis Assay kit (Thermo Fisher, UK) according to manufacturer's instructions, detailed in section 2.15. The fluorescence intensity of a linear dilution of fluorescent *E. coli* beads in assay buffer was measured to calculate approximate level of beads phagocytosed by macrophages.

### Gene Expression Analysis

Gene expression was assessed by TaqMan Gene Expression Assays (ThermoFisher Scientific) following mRNA isolation by innuPREP RNA Mini Kit (Analytikjena, Cambridge) and reverse transcription (Multiscribe, ThermoFisher Scientific) as per the manufacturer's guidelines. Expression of genes was determined using species‐specific probe sets by real‐time PCR on an QuantStudio 5 Real‐Time PCR System (Applied Biosystems, Warrington, UK) (**Table** **2**). mRNA abundance was normalized to either 18S. Data, obtained as Ct values and ΔCt determined (Ct target – Ct 18S or GAPDH), were expressed as arbitrary units (AU) using the following transformation: [AU = 1000 × (2^−Δct^)].

**Table 2:** Commercially available real‐time PCR primers and probes.

Target gene	Applied Biosystems reference number
*18S*	431943E
*CD163*	Hs00174705_m1
*GILZ*	Hs00608272_m1
*IL‐6*	Hs00985639_m1
*IL‐12A*	Hs01073447_m1
*TNFα*	Hs01113624_g1

### ELISA Analysis

Production of the pro‐inflammatory M1‐like and M2‐like cytokines within conditioned culture media were assessed using enzyme‐linked immunosorbent assay (ELISA). Human Uncoated ELISA Kits for TNFα, IL‐12 p70, and interleukin 10 (IL‐10) (Thermo Fisher Scientific, USA) were performed as per manufacturers protocol on Precoated Quantikine plates.

### Models of Polyarthritis

Procedures were performed under guidelines by the Animal (Scientific Procedures) Act 1986 in accordance with the project license (P51102987) and approved by the Birmingham Ethical Review Subcommittee (BERSC). The TNF‐tg model of chronic inflammatory polyarthritis, obtained courtesy of Professor George Kollias (BSRC Fleming, Athens), was maintained on a C57BL/6 background with ad libitum access to standard chow and drinking water.^[^ ^53^ ^]^ At day 36 of age, at the first onset of measurable polyarthritis, male TNF‐tg mice (bred and maintained in house, starting weight 19.72 g; SD 1.94 g) received intra‐articular knee injections (10 µL) into both hind legs. These consisted of a control saline injection into the left leg, and one of three treatments into the right leg (1. sheared hydrogel loaded with saline; 2. sheared hydrogel loaded with methylprednisolone sodium succinate (10 mg mL^−1^); 3. Saline containing methylprednisolone sodium succinate (10 mg mL^−1^)). Mice were scored for global disease activity, joint inflammation, mobility, and pain as previously described.^[^ ^54^, ^55^ ^]^ At day 58, serum was collected by cardiac puncture and tissues excised for analysis.

### Histological Staining and Immunohistochemistry

Both knee joints were assessed from six animals per intervention wing. Legs were dissected and fixed in 4% paraformaldehyde, paraffin embedded, and sectioned at 5 µm. Sections were stained with hematoxylin and eosin (H&E) or alcian blue 8GX (1% w/v in 3% glacial acetic acid) for histological analysis. Articular synovitis, pannus size at the humerus/ulna joint interface and cartilage area at the articular surface of the tibia were determined using Image J software as previously reported.^[^ ^45^, ^56^ ^]^ For quantification, the mean of three adjacent 10 µm sections cut from the center of the joint from six animals were assessed. Quantification of *GILZ* positive cells in a 50 µm^2^ area was determined by immunohistochemistry in pannus and bone marrow of all animals. Positively stained cells were analyzed using Image J software. Antigen retrieval was achieved by immersing sections in 10 mm citric acid at 95 °C for 30 min. The Gilz (anti‐Rabbit) Mouse/Human Polyclonal antibody (Invitrogen, PA587243, 1:4000) was used to probe for Gilz. The signals were detected using the ImmPRESS horseradish peroxidase (HRP) Universal PLUS Polymer Kit (Horse Anti‐Mouse/Rabbit IgG) (Vector labs, MP‐7800) as per manufacturer instructions. After performing the 3, 3′‐diaminobenzidine (DAB) peroxidase step the sections were counterstained with Mayers Hematoxylin and washed in Scott's water. Murine quadriceps muscles were embedded in paraffin and cut to 10 µm sections for histology. Samples were stained with hematoxylin and eosin prior to quantitative analysis of fiber size distribution using Image J software. Measurements were taken in three 200 µm^2^ regions of the vastus medialis for six mice per group.

### Immunofluorescence

The following primary antibodies were used for immunofluorescence staining of tissues: goat anti‐mouse podoplanin (R&D systems, AF3244,1 in 100), mouse anti‐mouse RANKL (Proteintech, Cat No: 66610‐1‐Ig, 1 in 100), and rabbit ant‐mouse TNF‐α (Proteintech, Cat. No: 80258‐6‐RR, 1 in 50). The corresponding secondary antibodies used respectively were as follows: AlexaFluor 488‐conjugated donkey anti‐goat IgG (Invitrogen, Cat. No. A‐11055), AlexaFluor 647‐conjugated goat anti‐mouse IgG1 (Invitrogen, A‐21240), and Dylight 594‐conjugated horse anti‐rabbit IgG (Vector Laboratories, DI‐1594‐1.5)). All secondaries were diluted 1 in 1000. Mouse FFPE tissue sections were dewaxed in xylene and rehydrated in 97% industrial denatured alcohol. Tissues were washed in water and then microwaved in preheated Tris‐based antigen retrieved solution (Vector Laboratories, Cat. No. H‐3301‐250ML) for 10 min. Tissues were outlined with a hydrophobic IMMedge Hydrophobic Barrier PAP pen (Vector Laboratories, Cat. No. H‐4000‐1‐PEN) washed in TBS. Tissues were then treated with mouse blocking reagent included in the M.O.M. staining kit (Vector Laboratories, Cat. No. MP‐2400) for 1 h. Sections were washed briefly in TBS and blocked in 2X casein solution (Vector Laboratories, Cat. No. SP‐5020‐250ML) diluted in TBS. Sections were then incubated with primary antibodies, diluted in casein solution, overnight at 4 °C. Tissues were washed in TBS and then incubated with corresponding secondary antibodies diluted in 2X casein, for 1 h at room temperature. Tissues were then washed in TBS and treated with TrueView autofluorescence quenching kit (Vector Laboratories, Cat. No. SP‐8400) for 5 min. Tissues were then rinsed in TBS to remove aggregates and then mounted with Vectashield Vibrance with DAPI (Vector Laboratories, Cat. No. H‐1800). Tissue were then imaged and fluorescence quantified using a Zeiss LSM 880 confocal microscope and analysis software.

### Statistical Analysis

All data were analyzed using IBM SPSS Statistics v28.0.1.0 (IBM Analytics, USA) and GraphPad Prism v5.03 and v9.5 (GraphPad Software, USA), with a P‐value of ≤ 0.05 considered to be statistically significant. Normality of data was confirmed using the Shapiro‐Wilk normality test. Significance of DEGs was assessed two tailed unpaired Student's t‐test or Mann‐Whitney U‐test, with Benjamini‐Horchbeg correction for false discovery rate at 5%. Data were analyzed using Student's t‐test, or one‐way ANOVA with post hoc Tukey's test or two‐way ANOVA with Tukey correction as appropriate. Experiments were carried out with sample sizes of n ≥ 3, defined as independent primary cell cultures from different donors, unless stated otherwise in figure legend. * denotes *P* ≤ 0.05, ** denotes *P* ≤ 0.01, *** denotes *P* ≤ 0.001 and **** denotes *P* ≤ 0.0001.

### Ethical Statement

Procedures were performed under guidelines by the Animal (Scientific Procedures) Act 1986 in accordance with the project license (P51102987) and approved by the Birmingham Ethical Review Subcommittee (BERSC). Fibroblasts were isolated from biopsies of matched skin, bone marrow, and synovium removed during total knee arthroplasty from consenting patients who fulfilled the 2010 ACR/EULAR criteria for rheumatoid arthritis following ethical approval (REC reference 14/ES/1044 & 071H12041).

## Conflict of Interest

Both L.M. Grover and R.J.A. Moakes are inventors of a patent describing the production of sheared gels and are founders in a company seeking to commercialize the technology (Healome Therapeutics Ltd).

## Author Contributions

C.A., C.M., S.S., S.D., D.K., and A.P. contributed to experimental delivery and data collection. R.J.A.M., L.M.G., K.R., and R.S.H. provided study management, data analysis, interpretation, and supervision. H.A. designed methods and performed chemistry for synthesis of steroid salts used in gels. A.F. and S.W.J. provided resources and expertise in primary culture models. All authors reviewed and edited the final version of the manuscript.

## Supporting information



Supporting Information

## Data Availability

The data that support the findings of this study are available from the corresponding author upon reasonable request.
