# Supplementary material for: Structured Polymers Enable the Sustained Delivery of Glucocorticoids within the Intra‐Articular Space
Source: Adv Healthc Mater. 2024 Dec 23;14(4):2403000. doi: 10.1002/adhm.202403000 (PMC11804841; doi:10.1002/adhm.202403000)
Supplement: Supplementary file 1 — Supporting Information [file ADHM-14-0-s001.docx]

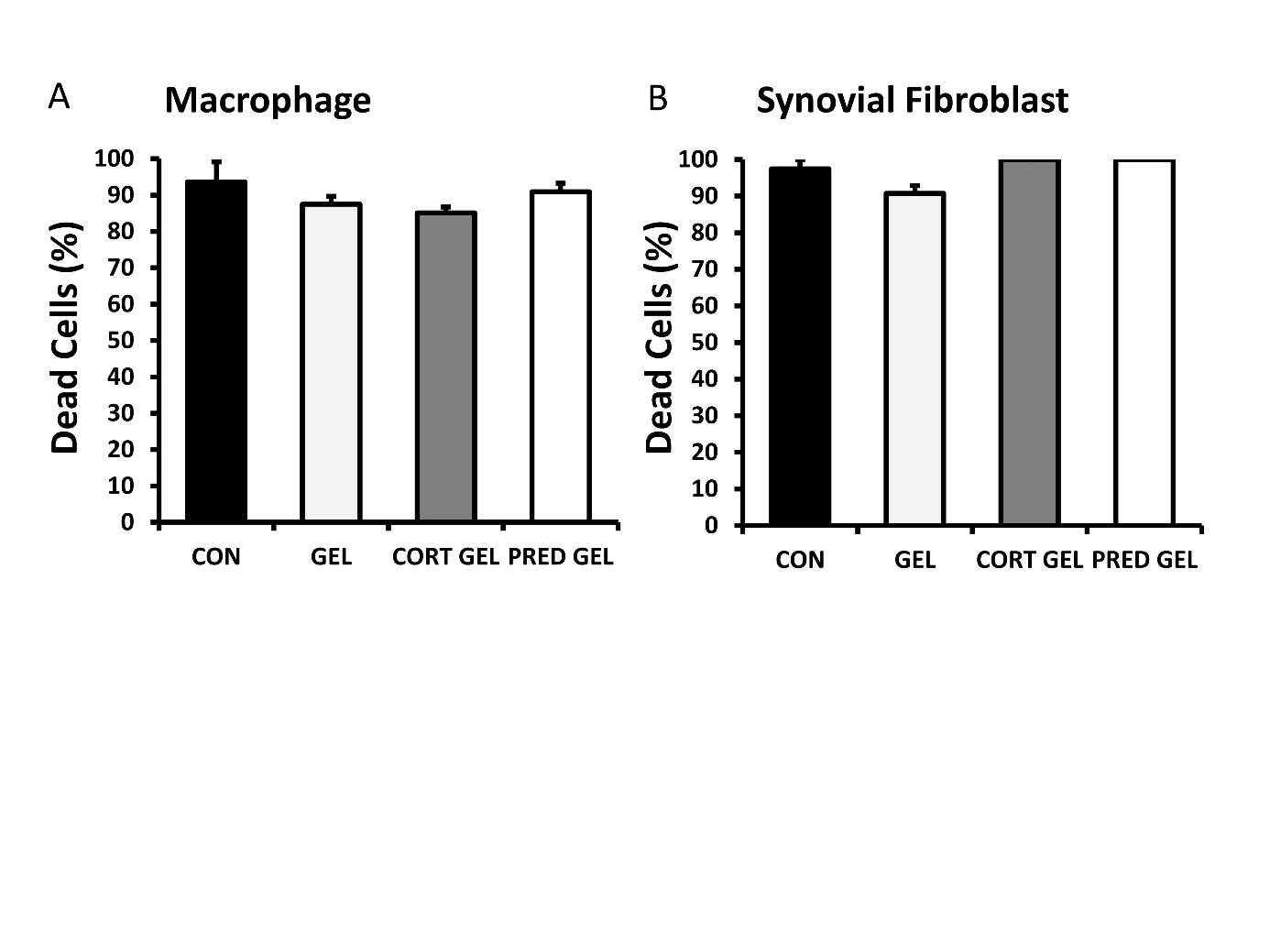


**Figure S1**: A-B, cytotoxicity determined by % of trypan positive staining cells in primary cultures of macrophage and treated with in primary human macrophages and synovial fibroblasts cultured with 25 µl of gellan sheared hydrogel (GSH) (1.5 % gellan (w/v), 10 mM NaCl,) containing either vehicle (Gel), 5 µM cortisol (Cort Gel) or methylprednisolone sodium succinate (Pred Gel) 10 mg/ml, relative to untreated control for 48 h. Data are presented as mean ± SEM of at least three primary cultures from three independent patient donors. Statistical significance was determined using one-way ANOVA with Tukey’s multiple comparisons test (* p≤0.05, **p≤0.01, *** p≤0.001).


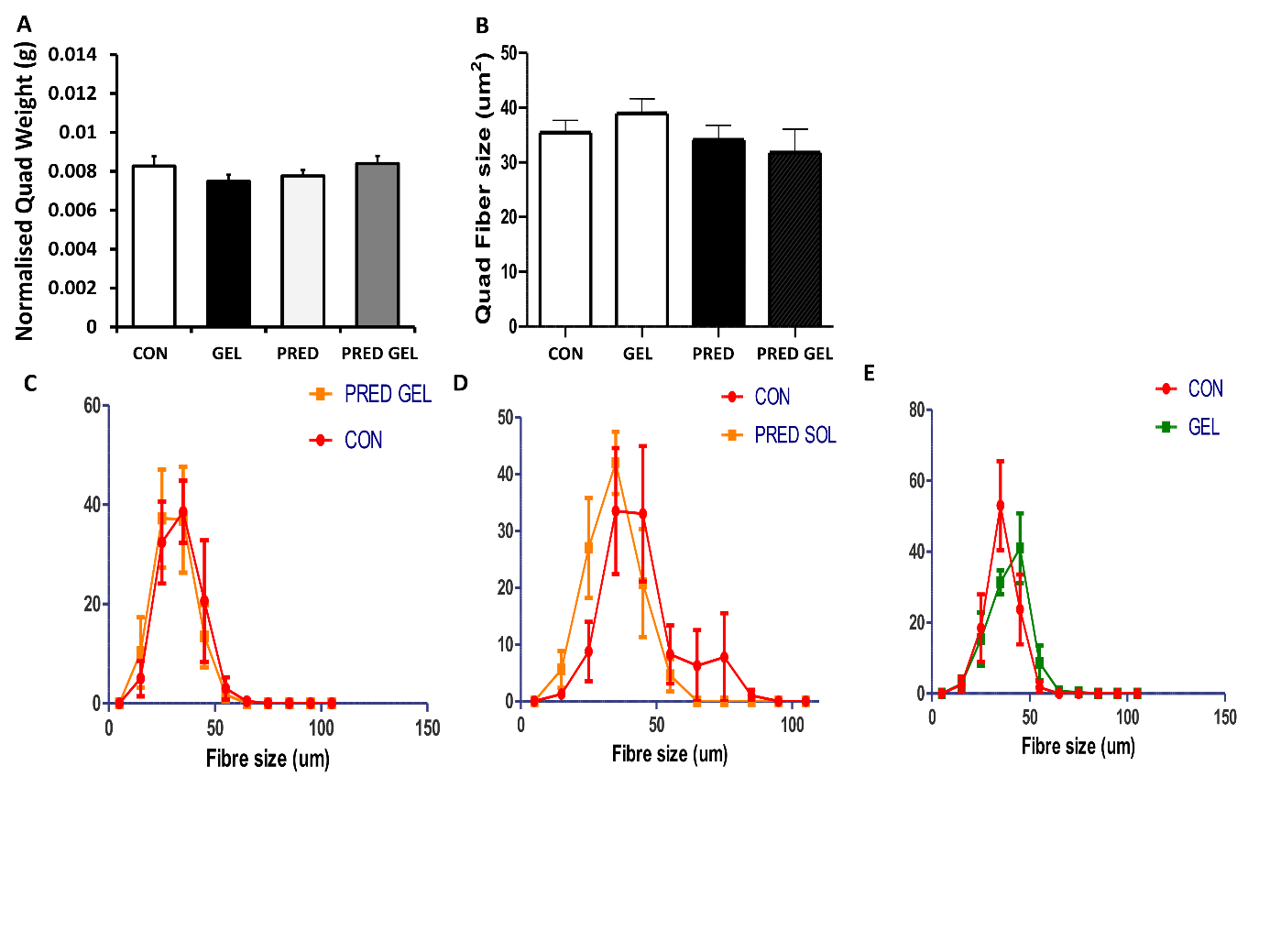


**Figure S2:** (a) Juxta-articular quadriceps muscle weights relative to total bodyweight and (b) average quadriceps muscle fiber cross sectional area (μm2) in TNF-tg, animals receiving intra-articular injection of either saline control (Con), Blank GSH (Gel: 1.5 % gellan (w/v), 10 mM NaCl), methylprednisolone sodium succinate (Pred: 10 mg/ml) or GSH loaded with methylprednisolone sodium succinate (Pred Gel: Gel: 1.5 % gellan (w/v), 10 mM NaCl, Pred 10 mg/ml). (c-e) Distribution of quadriceps muscle fiber cross-sectional area was determined using Image J in paraffin embedded sections in TNF-tg. Data are expressed as mean ± standard error of six animals per group. Statistical significance was determined using two-way ANOVA with a Tukey post hoc analysis. *P < 0.05, **P < 0.005, ***P < 0.001
